# The effectiveness of a Pilates exercise program during pregnancy on childbirth outcomes: a randomised controlled clinical trial

**DOI:** 10.1186/s12884-021-03922-2

**Published:** 2021-07-02

**Authors:** Nasim Yousefi Ghandali, Mina Iravani, Abdolhamid Habibi, Bahman Cheraghian

**Affiliations:** 1 School of Nursing and Midwifery, Ahvaz Jundishapur University of Medical Sciences, Ahvaz, Iran; 2 Reproductive Health Promotion Research Center, Midwifery and reproductive health Department, School of Nursing and Midwifery, Ahvaz Jundishapur University of Medical Sciences, Ahvaz, Iran; 3 Faculty of Sports Sciences, Shahid Chamran University of Ahvaz, Ahvaz, Iran; 4 Department of Biostatistics and Epidemiology, School of Health, Ahvaz Jundishapur University of Medical Sciences, Ahvaz, Iran

**Keywords:** Exercise during pregnancy, Pilates, Labor pain, Delivery outcome

## Abstract

**Background:**

Performing exercise with medium intensity has positive effects on the maternal health. The aim of this study was to investigate the effectiveness of Pilates exercise program during pregnancy on childbirth outcomes:

**Methods:**

This clinical trial study was performed on 110 primiparous women who were randomly divided into two groups of intervention (*n* = 55) and control (*n* = 55). The intervention group performed Pilates exercises from 26 to 28 weeks of gestation for 8 weeks while the control group did not do any exercise. Data collection tools included Visual Analog Scale (VAS), Mackey Childbirth Satisfaction Rating Scale, and a checklist including demographic and obstetrics information.

**Results:**

The results of the study showed that Pilates exercise during pregnancy significantly reduces the labor pain intensity, length of the active phase and second stage of labor and increases maternal satisfaction of the labor process (*p* < 0.05). Based on the Kaplan Meyer analysis, the mean whole length of labor was shorter in Pilates exercise group than in the control group (*P* = .004). There was no statistically significant difference between the two groups in terms of Episiotomy, type of delivery, first and fifth Apgar score of neonates (*p* > 0.05).

**Conclusion:**

According to the results of this study, Pilates exercise during pregnancy improved the labor process and increased maternal satisfaction of chidbirthprocess, without causing complications for the mother and baby. However, studies with larger sample sizes are recommended to prove the efficacy and safty of this practiceduring labor.

**Trial registration:**

**IRCT registration number:** IRCT20200126046266N1. **Registration date: 2020-05-02** (retrospectively registered).

## Background

The goal of obstetric care is to provide the right conditions for a safe delivery and make it a pleasant experience [1]. Labor pain is an unavoidable component of childbirth whose proper management, despite the great advances in midwifery, is still one of the major challenges related to women’s health [2]. The use of non-medical methods to reduce labor pain is compatible with obstetric management and most women opt for such methods [3].

On the other hand, prolonged delivery time is a clinical problem in modern midwifery that causes many problems for the mother and baby [4] such as increased maternal fatigue, induction, cesarean and instrumental delivery, uterine atony, maternal mortality as well as increased fetal distress, hypoxia, low Apgar score and ultimately fetal death [5].

Current evidence supports the initiation of exercise for sedentary women during pregnancy [6]. To date, no data have been reported on the dangers of moderate-intensity exercise for the mother or infant [7]. According to the latest guidelines women with low-risk pregnancies are encouraged to maintain or begin aerobic and progressive resistance training before, during, and after childbirth. Pregnant women should also have a clinical evaluation before starting exercise to make sure there is no medical reason to stop doing exercise [8]. A systematic review and meta-analysis in 2019 showed that in mothers with prenatal medical problems (chronic hypertension, type 1 diabetes and type 2 diabetes), prenatal exercise reduces the risk of cesarean section by 55% and does not increase the risk of adverse outcomes in mothers and infants (OR: 0.45; .95% CI, 0.22–0.95) [9].

Internationally, Pilates is considered a major exercise for improving physical, psychological, and motor functions [6]. This exercise includes a series of low-pressure exercise that build strength and flexibility throughout the body. When doing Pilates, adopting a standard breathing technique is very important and helps to activate the deep stabilizing muscles, especially the transversus abdominis in relation to the pelvis, which improves the strength of the pelvis and the trunk [10]. Pilates movements can be performed according to the physiological changes of pregnancy [6].

Regular training has been shown to strengthen the pelvic floor muscle and increase its structural function [10]. Pelvic floor muscle contraction exercises are part of modern Pilates [11]. Pelvic floor exercises have been shown to prevent prolonged second stage of labor labor in about one in eight women during pregnancy [12].

In pregnancy Pilates, Pilates diaphragmatic breathing technique, which helps the mother prepare for childbirth. The imaging technique used in Pilates prepares the mother to use this technique during labor [13]. Improving the ability of the trunk and pelvic floor muscles, flexibility and proper breathing in Pilates may facilitate the delivery process, but since limited randomized controlled trials have been conducted on the effect of Pilates during pregnancy on the delivery process, Therefore, the present study was conducted to examine the effect of Pilates on the childbirth process and its outcomes in primiparous women.

## Method

### Setting

The present study was performed from winter 2020 to the end of May 2020 in selected health centers and the maternity ward of 22 Bahman Hospital of Masjed Soleiman, Southwest of Iran.

### Design

This study was a single-blind randomized clinical trial on 110 pregnant women referring to health centers and the maternity ward in Masjed Soleiman. Before commencement of the study, it was approved by the Ethics Committee of Ahvaz Jundishapur University of Medical Sciences, and the participants gave informed consent to participate in the study. All of these participants were pregnant women under the care of health centers and gynecologists. This clinical trial was retrospectively registered at the Clinical Trials Registration Center (Reference No.: IRCT20200126046266N1, retrospectively registered).

### Inclusion and exclusion criteria

Inclusion criteria included age between 18 and 35 years, first pregnancy, single pregnancy, gestational age between 26 and 28 weeks, normal Body mass index, and willingness to participate in the study. Exclusion criteria included diseases such as pregnancy hypertension, gestational diabetes, heart problems, skeletal problems, medical prohibition to do exercise during pregnancy, absence from more than two sessions in the exercise programs and withdrawal from the study.

### Randomization

The statistical population included all primiparous women referring to Masjed Soleiman health centers. Our method was convenience non probability sampling. Thus, from the beginning of the study, all women who had inclusion criteria and no exclusion criteria were included in the study, and this process continued until reaching the final sample size. Participants were registered by the researcher. These women were randomly assigned into two groups of control (*n* = 55) and intervention (*n* = 55) using the permuted block randomization method (blocks of six with a ratio of 1: 1). Participants were randomly selected using envelopes sealed by a health center staff member who was unaware of the nature of the study. The researchers and instructors involved in the training and the evaluators had no role in the randomization process..The study was single-blind. That is, the people evaluating the results of the study (midwifery staff working in the maternity ward of the hospital and the statistician) were blind to group assignment. Due to the nature of the study, blinding of the pregnant mothers to group allocation was not possible.

### Intervention

In the intervention group, a Pilates exercise program, which was tailored to the conditions of pregnant women, was developed in coordination with a Pilates instructor and was performed twice a week for 8 weeks. Participants in the intervention were asked to choose a number from 6 to 20 in terms of the intensity of the effort to perform the exercises. Exercises started with light intensity and after two weeks of adjustment, the intensity increased. Because mothers’ physical strength during pregnancy is different and the intensity of exercise may be moderate for one mother but severe for another, causing severe sweating and fatigue. Of course, the intensity of the exercises had to be not more than 14 according to The Borg Rating of Perceived Exertion (RPE) [8], and if the exercise was estimated to be intense for the participant, she would not perform that exercise and would replace it with a lighter one. Before starting to exercise, participants were instructed to stop exercising if their perceived exertion increases above 14 on a scale from 6 to 20. After exercising all participants were asked to rate their perceived exertion on a scale from 6 to 20.

During the program, groups of 9 to 10 pregnant women were present to ensure a suitable space for exercise. Exercises with balls and fabric bands were also used during the sessions. Specific pelvic floor exercises, including contraction of the pelvic floor muscles, were performed intermittently with 5 to 10 repetitions. Each session included a warm-up phase (5 min), pregnancy specific Pilates exercises (25 min), and the phase of returning to a relaxed state (5 min) in which relaxation techniques were performed. After the exercise, the mothers would lay on their left side for 30 min and rested. The first 10 to 12 sessions of training were held in the presence of a sports coach with groups of 9 to 10 people in the gym. However, the last 4 to 6 training sessions were held at home under the supervision of an instructor due to the spread of the coronavirus. Participants’ adherence to the exercise regime was monitored through a daily exercise booklet. Once every two weeks, the control group received routine pregnancy advice over the phone and engaged in their daily activities and did not participate in any regular exercise program.

During and after childbirth care in both intervention and control groups was performed according to the standard and routine protocols of the hospital. During delivery, a checklist modified according to the conditions of each of the studied groups was completed.

### Measurement instrument

Four instruments were used in the present study, including a two-section checklist, Borg Rating of Perceived Exertion (RPE), Visual Analog Scale (VAS), and Mackey Childbirth Satisfaction Rating Scale.

In this study, a checklist consisting of two sections was used to collect the required information. In the first section, the demographic information of the studied subjects was recorded. This information included the mother’s age, level of education, occupation, gestational age, and body mass index. In the second section of the checklist, obstetric information with regard to the research objectives was recorded. This included the severity of pain in dilatations of 3, 6, 8 and 10 cm, length of labor, type of delivery, oxytocin consumption, use of episiotomy, first- and fifth-minute Apgar score, and the mothers’ satisfaction with the childbirth experience. Intensity of labor pain was measured by Visual Pain Scale (VAS) consisting of a horizontal line 10 cm long with a score of zero for no pain and a score of 10 for maximum pain. To measure maternal satisfaction with childbirth, Mackey Childbirth Satisfaction Rating Scale was used, which included 22 questions in 4 sub-scales: the mother’s satisfaction with her performance, with the midwives’ performance, with infant status, and overall satisfaction with labor and childbirth experience. These are measured using a 5-point Likert scale including “very satisfied” (score 5), “satisfied” (score 4), “neither satisfied nor dissatisfied” (score 3), “dissatisfied” (score 2), and “very dissatisfied” (score 1). The total score obtained from this tool varies between 110 and 22. Scores between 22 and 55 are classified in the dissatisfied group, 88–56 in the desired satisfaction group, and 89 and above in complete satisfaction group. The intensity of exercise was assessed and recorded by the Borg Rating of Perceived Exertion (RPE). This scale consists of a 15-degree vertical line graded along its axis from 6 to 20 degrees. For moderate exercise, the American College of Obstetrics and Gynecology recommends the perceived severity of 14–13 (somewhat hard) to be used [11].

Visual pain measuring tool is the most widely used pain measuring tool in the world. In several studies abroad, the validity and scientific reliability of this tool has been confirmed and in Iran, the reliability of this scale has been confirmed with a correlation coefficient of r = 0.88 [14].

McKay Delivery Satisfaction Questionnaire has already been translated into Persian and used in Iran and its validity has been confirmed by content validity method and its reliability has been confirmed by calculating Cronbach’s alpha (α = 0.92) [15].

To calculate the sample size, the two-ratio comparison formula was used:

$$ n=\frac{{\left({z}_{1-a/2}+{z}_{1-\beta}\right)}^{2\ast}\left[{p}_1\;\left(1-{p}_1\right)+{p}_2\;\left(1-{p}_2\right)\right]\;}{{\left({p}_1-{p}_2\right)}^2} $$

Sample size was calculated based on the number of episiotomies after 8 weeks of Pilates exercise as in Rodriguez study. Where α = 0.05, β = 0.1 and based on the results of previous similar studies [16], we assumed P 1 = 0.1, P 2 = 0.42. Therefore, the initial sample size was considered 44, and taking into account the 20% attrition rate, the final sample size was estimated to be 55 in each group (110 people in total). Primary outcome included episiotomy. Secondary outcomes included severe labor pain, length of the first stage of labor, the length of the second stage of labor, and the type of labor, the need for oxytocin induction, first- and fifth-minute Apgar scores, and delivery satisfaction.

### Statistical analysis

To analyze the data, descriptive statistical methods including frequency distribution tables, chart of central indicators and appropriate distribution were used to describe the study data. Quantitative data normality was checked by Kolmogorov-Smirnov test. The relationship between qualitative variables was assessed using Chi-square test. Within group relationship between quantitative variables was examined by paired t-test, and for between group examinations of quantitative variables, independent t-test and Mann-Whitney test were used. Because the distribution is normal, to describe 5 variables of age, gestational age at the beginning of the intervention, BMI, length of pregnancy, and weight of the baby, we used mean and standard deviation. For statistical tests, t-test was used. To describe other quantitative variables such as the lengths of the first and second stages of labor, pain intensity, first- and fifth-minute Apgar scores and oxytocin use, the middle (interquartile range) was used, and nonparametric tests such as the Mann-Whitney test were used. Kaplan-Meier test and Cox regression analysis were used for the entire duration of labor. In order to control possible confounding factors, analysis of covariance was used if necessary. Data analysis was performed with SPSS version 25.

## Results

**Fig. 1:**
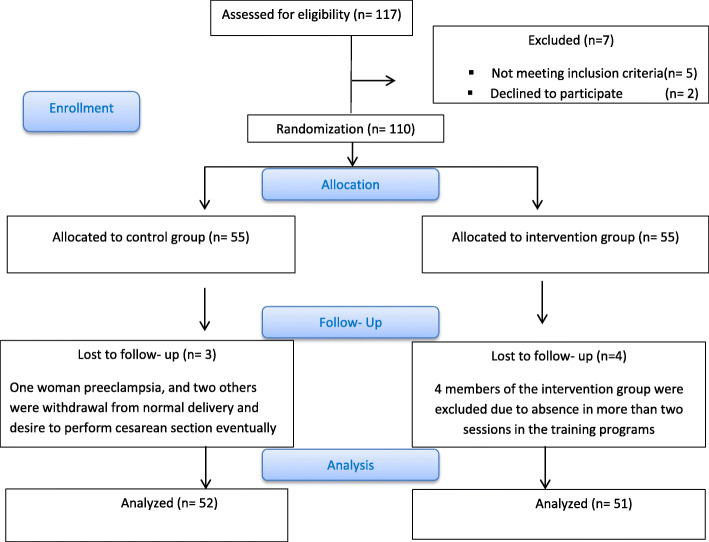
Flowchart of the progress through the phases of the trial

In the present study, out of the 110 subjects who entered the study, 4 members of the intervention group were excluded from the study due to absence in more than two sessions in the training program, One woman in the control group was excluded from the study due to preeclampsia at the 34th week of gestation, and two others were excluded from the study due to withdrawal from normal delivery and desire to perform cesarean section Eventually, the final sample size was 103 (51 in the intervention group and 52 in the control group). Flowchart of the study is shown in Fig. 1.

According to Table 1, the mean age of the participants in the study group was 25.16 ± 4.41 years, and that in the control group was 23.81 ± 4.30 years. The mean gestational age at the beginning of the study was 26.71 ± 0.78 weeks in the intervention group and 26.67 ± 0.73 weeks in the control group. The mean body mass index in the first 12 weeks of pregnancy was 22.71 ± 1.57 kg/m2 in the intervention group and 22.38 ± 1.52 kg/m2 in the control group. According to the independent t-test, there was no significant difference between the two groups in terms of gestational age at the time of enrollment and body mass index at the beginning of the study. The education level of most of the women (81.55%) in the study was diploma or higher. As far as occupation was concerned, 96 (93.2%) of the participants in the study were not employed, there was no significant difference between the two groups in terms of education level and occupational status.

**Table 1:** Mean and frequency distribution of demographic characteristics

Variable		Control group (*n* = 52)	Intervention group (*n* = 51)
		Mean ± SD	Mean ± SD
Age		23.81 ± 4.30	25.16 ± 4.41
BMI (kg/m^2^)		22.38 ± 1.52	22.71 ± 1.57
Gestation age (weeks) at study entry		26.67 ± 0.73	26.71 ± 0.78
		Frequency (percentage)	Frequency (percentage)
Educational attainment	Did not finish high school	9 (17.3)	10 (19.6)
	Finished high school	24 (46.2)	22 (43.1)
	University education	19 (37.3)	19 (36.5)
Employment status	Employed	3 (5.8)	4 (7.8)
	Not employed	49 (94.2)	47 (92.2)

There was no statistically significant difference in the mean pain intensity in the 3-cm dilation (latent phase) (*p* = 0.46). As far as pain intensity in 6-cm dilation (*p* = 0.000, z = 4.531), 8-cm dilation (p = 0.000, z = 5.258) and full dilation (p = 0.000, z = 4.675) as measured by VAS was concerned, there was a statistically significant difference. (Table 2; Fig. 3).

**Table 2:** The comparison of Mean ± SD OR Frequency (%) of childbirth outcomes in control and intervention groups

Variable		Control group (*n* = 52)	Intervention group (*n* = 51)	*P*-value*	Cohen’s d
		Mean ± SD	Mean ± SD		
Pain intensity at 6 cm dilation based on VAS		6.14 ± 1.07	5.04 ± 0.99	0.000	1.05
Pain intensity at 8 cm dilation based on VAS		7.46 ± 1.16	6.20 ± 0.87	0.000	1.23
Pain intensity at full cervical dilation based on VAS		8.51 ± 1.14	7.44 ± 0.81	0.000	1.07
Duration of active phase of labor (min)		164 ± 99.81	110 ± 70.94	0.004	0.61
Duration of second stage of labor (min)		50.36 ± 38.59	33.49 ± 24.51	0.043	0.52
1 min APGAR score		8.58 ± 0.96	8.86 ± 0.40	0.074	0.39
5 min APGAR score		9.88 ± 0.38	9.94 ± 0.24	0.468	0.18
Administered oxytocin (mL)		620.19 ± 783.73	396 ± 469.02	0.160	0.36
Variable		Frequency (%)	Frequency (%)	*P*-value**	
Episiotomy	Yes	37 (88.1)	32 (71.1)	0.051	
	No	5 (11.9)	13 (28.9)		
Type of delivery	Vaginal	42 (80.8)	45 (88.2)	0.296	
	Cesarean	10 (19.2)	6 (11.8)		
Satisfaction with delivery	Unsatisfied	6 (14.3)	1 (2.2)	0.000	
	Satisfied	29 (69.0)	19 (42.2)		
	Very satisfied	7 (16.7)	25 (55.6)		

The symbols * and ** are the values based on paired comparisons (the independent t-test/ Mann-Whitney test) and chi-squared test, respectively

The difference between the intervention and control groups in terms of the length of the active phase of labor, a statistically significant difference was observed (*P* = 0.004, z = 2.882). The mean length of the second stage of labor in the intervention group was 33.49 ± 24.51 min while it was 50.36 ± 38.59 min in the control group, there was a statistically significant difference between the two groups (*P* = 0.043, z = 2.026,) in this regard (Table 2; Fig. 3)..

**Fig. 2:**
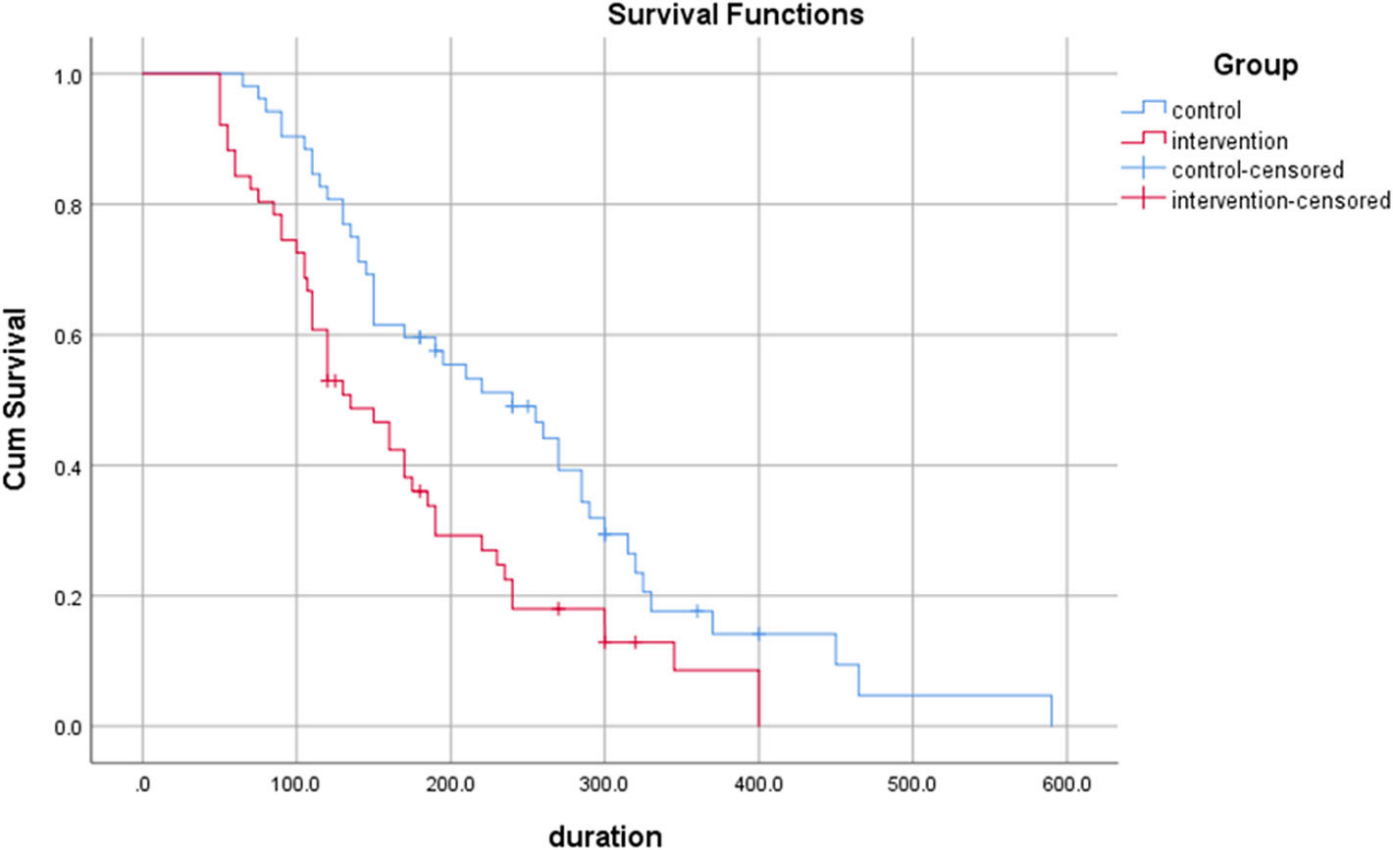
Total labor duration in control and intervention groups

Based on the Kaplan Meyer analysis, the mean whole length of labor was 170.42(95% CI: 140.63–200.22) in the intervention group vs. 247.54 (95% CI: 207.77–287.32) in the control group (*P*-value = .004). No cases of instrumental delivery were observed in the two groups, and cesarean deliveries were censored from the above analysis (Table 3; Fig. 2).

**Table 3:** The comparison duration of labor^**^ in control and intervention groups based on _Kaplan Meier survival analysis_

Group	Mean	Std. Error	95% Confidence Interval		Chi-square	*P*-value^*^
			Lower Bound	Upper Bound		
control	247.54	20.29	207.77	287.32	8.41	.004
intervention	170.42	15.20	140.63	200.22		

*Log-Rank Test; **the sum of the duration of the active phase and the second stage of labor

According to Cox regression analysis, body mass index has an increasing effect on the average total length oflabor, which is statistically significant (β = .287, *P*-value <.001) (Table 4).

**Table 4:** The effect of maternal body mass index on the duration of labor^**^ based on Cox model

Variable	Beta	Std. Error	95% Confidence Interval		Hazard Ratio	z	*P*-value^*^
			Lower Bound	Upper Bound			
BMI	.287	.082	.126	.448	1.33	3.50	<.001

*The duration of labor considering the maternal body mass index was analyzed by using Cox regression analysis; **the sum of the duration of the active phase and the second stage of labor

Also, Details of the comparison of median (25th percentile, 75th percentile) of childbirth outcomes in control and intervention groups are presented in Table 5.The mean of oxytocin consumption to increase uterine contractions during labor was 396.08 ± 469.02 cc in the intervention group and 620.19 ± 783.73 cc in the control group, and there was no statistically significant difference between the two groups (*P* = 0.160, z = 1.403) (Table 2).

**Table 5:** The comparison of median (25th percentile, 75th percentile) of childbirth outcomes in control and intervention groups

group Variable	Percentiles (control group)			Percentiles (intervention group)		
	25	50	75	25	50	75
Administered oxytocin (mL)	125	350	700	0	300	500
Duration of active phase of labor (min)	100	140	240	80	105	150
Duration of second stage of labor (min)	20	37	63	15	30	40
Pain intensity at 6 cm dilation based on VAS	5	6	7	4	5	6
Pain intensity at 8 cm dilation based on VAS	7	8	8	6	6	7
Pain intensity at full cervical dilation based on VAS	8	9	9	7	7	8
1 min APGAR score	8.2	9	9	9	9	9
5 min APGAR score	10	10	10	10	10	10

**Fig. 3:**
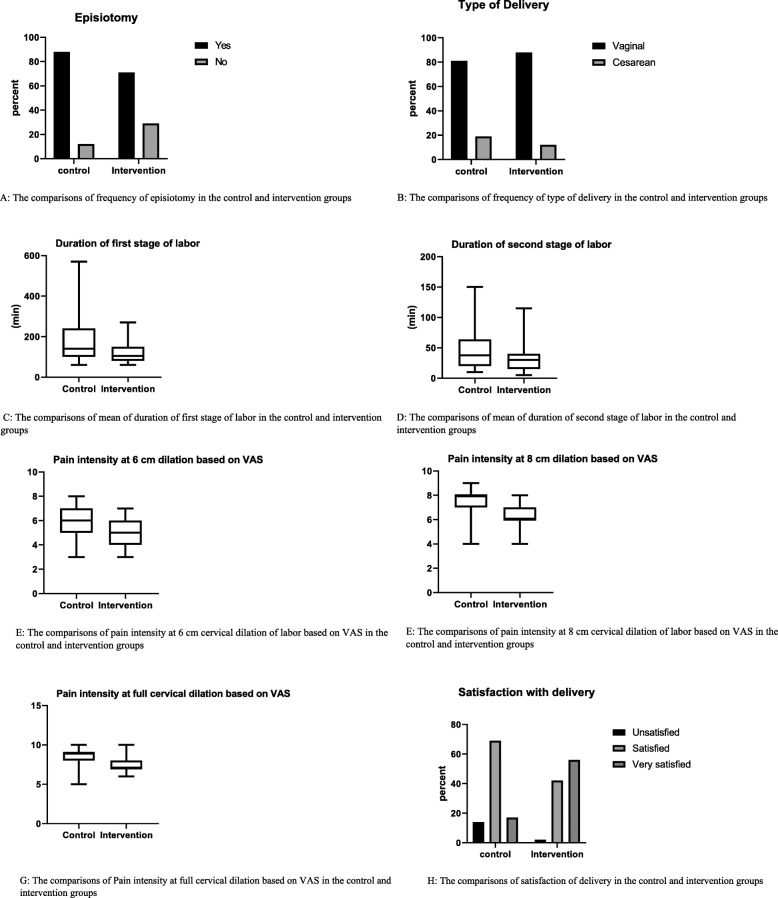
The comparison of Mean ± SD OR Frequency (%) of childbirth outcomes in control and intervention groups

Episiotomy was performed on 71.1% (95% CI: %55.7-% 83.6) of the women in the intervention group and on 80.1% (95% CI: %74- %96) of women in the control group, and, there was no statistically significant difference between the groups (*P* = 0.051, Df = 1) (Table 2; Fig. 3).

Also, (88.2%)(95% CI: %76 to %96) women in the intervention group and 80.8%(95% CI: %68 to %.90) women in the control group had a normal delivery, while 11.8% (95% CI: %044 to %90) women in the intervention group and 19.2%(95% CI: %096 to %33) women in the control group had emergency cesarean sections, there was no statistically significant difference between the two groups in terms of the type of delivery (*P* = 0.296, Df = 1) (Table 2; Fig. 3).

The mean first Apgar score of neonates in the intervention group was 8.86 ± 0.40 and 8.58 ± 0.96 in the control group, and there was no statistically significant difference between the two groups in this regard *P* = 0.074, z = 1.784). Also, the mean fifth-minute Apgar score of neonates was 9.94 ± 0.24 in the intervention group and 9.88 ± 0.38 in the control group, which were not significantly different (*P* = 0.468, z = 0.725). Also, according to Table 6, as far as Apgar score was concerned, 100% of the neonates in the intervention group and 94.2% of the neonates in the control group had a first-minute Apgar score of 7–10, while the fifth-minute Apgar score of all infants was in the range of 7–10 (Table 2; Fig. 3).

**Table 6:** Frequency distribution of 1 min and 5 min APGAR scores for control and intervention groups

Variable		Control group (*n* = 52)	Intervention group (*n* = 51)	*P*-value
		Frequency (percentage)	Frequency (percentage)	
1 min APGAR score	0–3	0 (0)	0 (0)	0.243
	4–6	3 (5.8)	0 (0)	
	7–10	49 (94.2)	51 (100)	
Variable		Control group (*n* = 52)	Intervention group (*n* = 51)	*P*-value
		Frequency (percentage)	Frequency (percentage)	
5 min APGAR score	0–3	0 (0)	0 (0)	1
	4–6	0 (0)	0 (0)	
	7–10	52 (100)	51 (100)	

In Table 2, there was a statistically significant difference between the two groups in terms of maternal satisfaction with the delivery process (*P* < 0.001, df = 1) (Table 2; Fig. 3).

## Discussion

The aim of this study was to investigate the effect of Pilates exercise on the outcomes of childbirth in primiparous women. According to the results of the present study, Pilates had a positive effect on the severity of labor pain in the active phase, the length of the active phase and second stage of labor, and maternal satisfaction with childbirth. Nevertheless, there was no association between Pilates exercise during pregnancy and need to oxytocin to increase labor pain, episiotomy, type of delivery, and first- and fifth -minute Apgar scores. Also, the results of Cox regression analysis showed that the duration of labor was longer in women with higher body mass index. In this analyses, BMI was included as a possible confounder.

Rodríguez et al. showed the beneficial effects of an 8-week Pilates program during pregnancy on the better control of labor pain (reduced epidural anesthesia). Studies highlight that regular exercise during pregnancy strengthens the pelvic floor muscles to help relieve pain and reduces the need for epidural anesthesia during labor [16]. In studies of Aktan and Sarpkaya Güder et al., Pilates exercises during pregnancy also helped women suffer less pain during childbirth. Studies show that thanks to diaphragmatic breathing, women doing Pilates during their pregnancy can better get along with uterine contractions and prenatal pain, have an easier childbirth, and experience less labor pain [13].

In our study, the use of Pilates exercise during pregnancy could reduce the length of labor. In this regard, and in line with our study, in studies by Bolanthakodi, Barakat and Gehan, the length of labor in the exercise group was shorter than that in the control group [17–19]. Melzer also concluded that physical activity can reduce the length of the second stage of labor compared to no physical activity, but the first stage of labor is not affected by it [20]. In a study by Perales, in contrast, physical activity during pregnancy caused the first stage to be shorter but had no effect on the length of the second stage of labor [21]. A systematic review conducted in 2019 showed that exercise did not affect the duration of labor [22], the results of Salvesen study also showed that regular exercise did not affect the duration of labor compared to women in the control group [23]. Diaphragmatic breathing and proper exhalation techniques to strengthen the diaphragm can help the uterine muscles function properly during labor and help in pushing the baby out. Furthermore, in the studies of Aktan and Sarpkaya Güder, Pilates exercises during pregnancy had no effect on the length of labor [13]. The results of our study showed that doing exercise during pregnancy reduces the complications associated with prolonged and painful labor by reducing the length of labor.

Although in the present study Pilates did not significantly affect the need for oxytocin to augmentation of labor pain, the need for infusion of oxytocin was lower in the intervention group than that in in the control group. Two studies conducted by Salvesen were consistent with our study [12, 23]. A systematic review by Davenport et al. on 113 studies also showed that exercise had no effect on induction of labor [22].

The results of the present study showed that although fewer cases of episiotomy were performed in the intervention group than in the control group, this difference was not significant, which is in line with Salosan’s and Melzer’s studies [20, 23]. On the other hand, the results were inconsistent with those of Rodríguez’s study in which a significant difference was observed in the reduction of episiotomy in the Pilates group compared to the control group [16]. It seems that primiparity, short perineal length, race, and induction of pain with oxytocin are among the factors that increased the incidence of episiotomy in the present study. In fact, it can be concluded that episiotomy is not affected by Pilates exercises alone, and other factors such as race, length of Perineum, parity, induction of labor, fetal weight, stable posterior occipital position, breech delivery, etc. also contribute to performing episiotomy.

The results of the present study showed no effect of Pilates exercise during pregnancy on the type of delivery. Studies of Rodríguez and Sarpkaya Güder showed a statistically significant difference in the type of delivery between Pilates and control groups, with the number of normal deliveries being higher in the Pilates group [13, 16]. However, in the study of Aktan, Pilates during pregnancy had no effect on the type of delivery, which was consistent with our results [13]. Davenport in a systematic review with a review of 52,858 women Do not get a difference in the rate of cesarean section with exercise [22]. The main difference between the intervention groups was in the intensity of exercise. It seems that the evidence supporting the role of exercise in reducing the rate of primary cesarean section is due to differences in the amount of exercise [24]. Barakat did not find any difference between the control group and active people who did only light exercise 3 days a week during pregnancy [25]. A review and meta-analysis showed that women who did aerobic exercise for 30 to 60 min 2–7 times a week had a significantly reduced risk of cesarean delivery [26]. The results of this study showed that moderate-intensity Pilates exercise twice a week alone had no effect on increasing the rate of natural childbirth in primiparous women, and that most young primiparous women had a better chance of having a normal delivery. Also, due to the cancellation of face-to-face sessions following the outbreak of the coronavirus and doing the exercises at home, it seems likely that doing exercise in the presence of a coach will yield different results.

In a study on the effect of exercise during pregnancy on labor outcome, Price and Melzer found that exercise during pregnancy had no effect on neonatal Apgar score [20, 24], which is consistent with the present study. In contrast, in the studies of Aktan and Sarpkaya Güder, Pilates exercises during pregnancy caused a statistically significant difference between the intervention and control groups in terms of the first- and fifth-minute Apgar scores [13], which is inconsistent with the present study. One of the reasons for the no effect of Pilates on Apgar score could be attributed to the fact that in the study environment, if there are risk factors that cause low Apgar score of the baby, cesarean delivery is performed quickly as an emergency, which is one of the reasons for reducing the cases with low Apgar score. In the present study, no adverse effects on neonatal outcomes were observed.

Consistent with the present study, in a study by Sarpkaya Güder, compared with the control group, women who did Pilates exercises during pregnancy felt more secure during delivery, coped better with labor pain, experienced fewer problems in the delivery process, and were generally more satisfied with the delivery experience [13]. In the study of Bolanthakodi, doing yoga exercises during pregnancy made mothers more satisfied with the delivery process [17]. Navaz also showed that controlling labor pain by exercises during pregnancy (without using epidural) can improve the mother’s experience of delivery and have long-term effects on her subsequent pregnancies [3]. In our study, Pilates also had an effect on maternal satisfaction with childbirth. According to the results and according to the participants, reduced length of labor, reduced intensity of labor pain, and the use of breathing techniques and relaxation during labor (which were learned in exercise sessions during pregnancy) will eventually make the experience of delivery easier and increase the level of women’s satisfaction with the delivery process.

The present study had several strengths. First: One of the strengths of this study is that the selection of primiparous women prevented the influence of the individual’s experience of previous childbirth on the study results. Second: the lack of concomitant use of medications to reduce labor pain made the role of prenatal exercise in reducing the severity of labor pain more realistic. Third: We used of Cox regression analysis and Kaplan Meier survival analysis for calculate the overall length of labor. Survival analysis is a useful procedure when a number of confounding factors effect on the results of the study. In this method of data analysis, these factors are censored.

One of the limitations of the present study is the poor mental state of pregnant women during exercise due to the spread of Covid 19 virus. There are also other important limitations. For instance, only low-risk and nulliparous pregnant women were included in this study, so the present findings cannot be generalized to high-risk pregnancies as well as multiparous mothers. Also, the lack of exercise in the control group left unanswered the question of whether a Pilates exercise program could prove more valuable in the pregnancy and delivery process compared to other sports. Finally, an intention to treat analysis was not possible as study outcomes could not be assessed in women who had Ceserean sections.

## Conclusion

According to the results of this study, Pilates exercise during pregnancy is a safe method to reduce the length of the active phase and second stage of labor, reduce labor pain, and increase maternal satisfaction with the childbirth process. However, Pilates exercise did not significantly reduce the need to episiotomy and cesarean section. It was not possible for us to assess outcomes such as episiotomy and duration of the active phase and second stage, as well as the severity of pain during labor in some mothers due to cesarean section. Therefore, the results this study should be interpreted with caution. Although in this study performing these exercises during pregnancy did not cause side effects for the mother and baby, more detailed studies with a larger sample size are needed to prove the effectiveness and safety of this exercise during pregnancy. In future studies, it is recommended that the Pilates exercise be performed from the beginning of the second trimester and continue for more than 8 weeks in pregnant women. It is also suggested that, the effect of this exercise on multiparous pregnant women be investigated.

## Data Availability

The datasets generated and/or analysed during the current research are not publicly available as individual privacy could be compromised but are available from the corresponding author on reasonable request.
